# Supplementary figures and images for: Determinants of gestational weight gain during pregnancy in a multiethnic UK-based population: Findings from the Born in Bradford cohort study
Source: PLoS One. 2025 May 23;20(5):e0323278. doi: 10.1371/journal.pone.0323278 (PMC12101682; doi:10.1371/journal.pone.0323278)

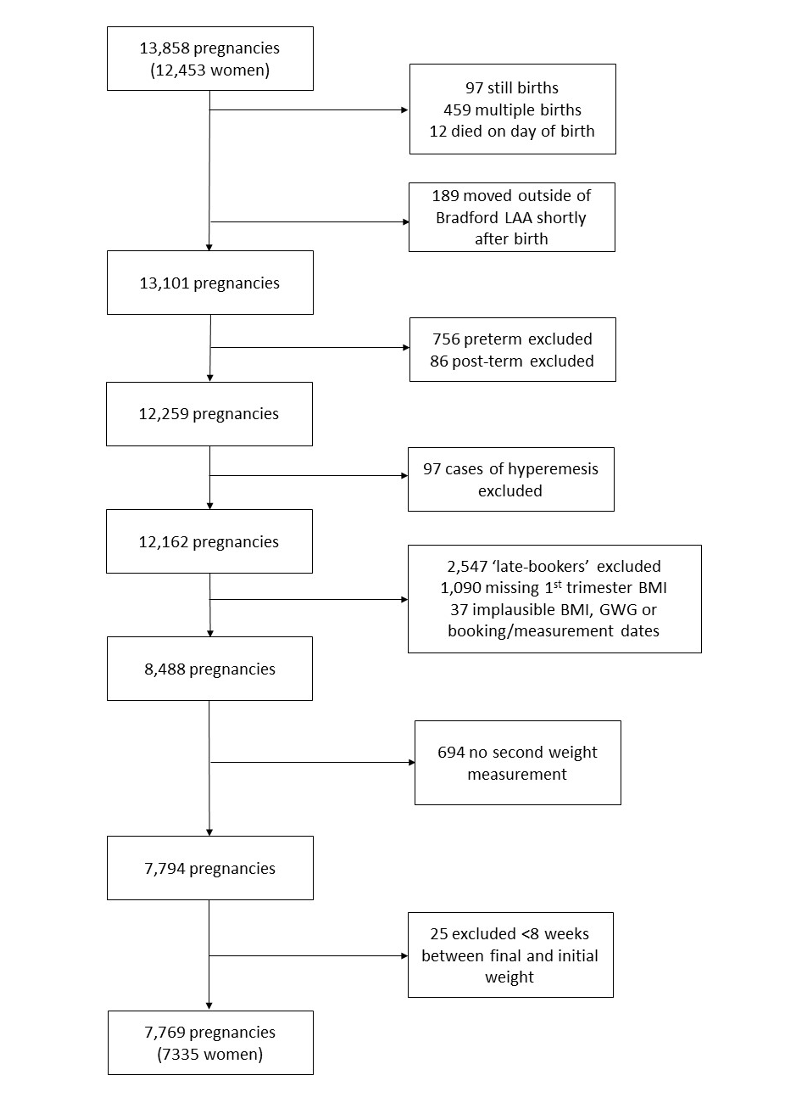

Supplement: S1 Fig — (TIF) [file pone.0323278.s001.tif]

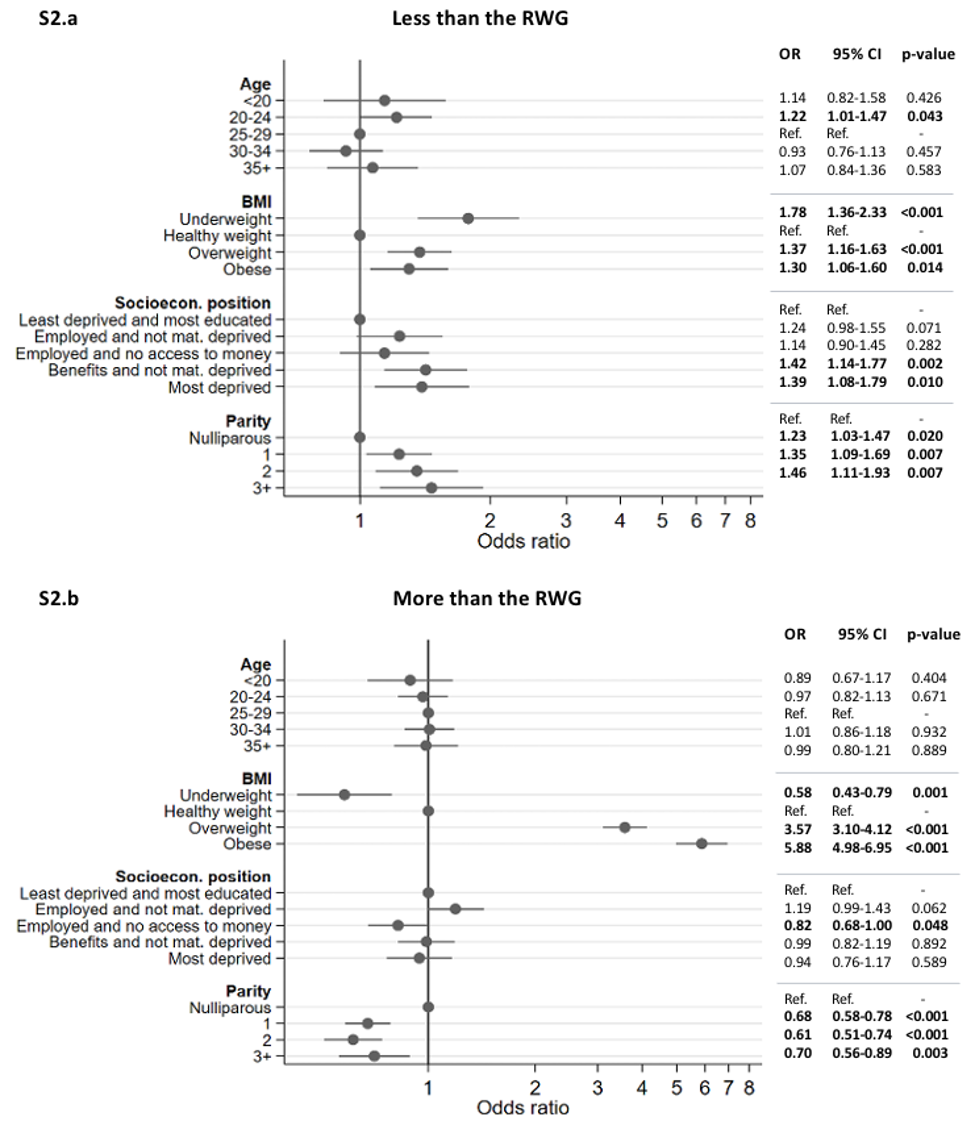

Supplement: S2 Fig — (TIF) [file pone.0323278.s002.tif]

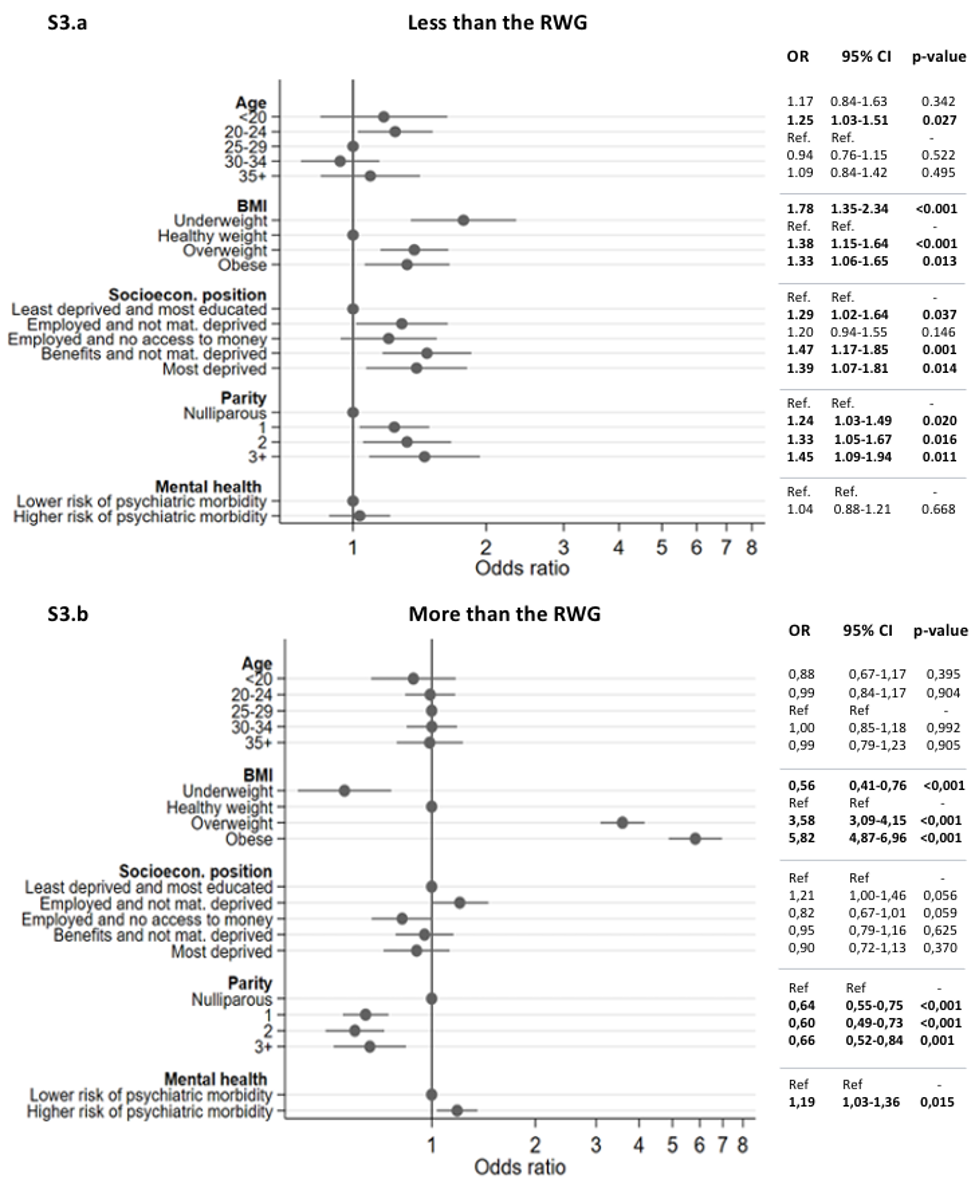

Supplement: S3 Fig — Results adjusted for ethnicity. (TIF) [file pone.0323278.s003.tif]

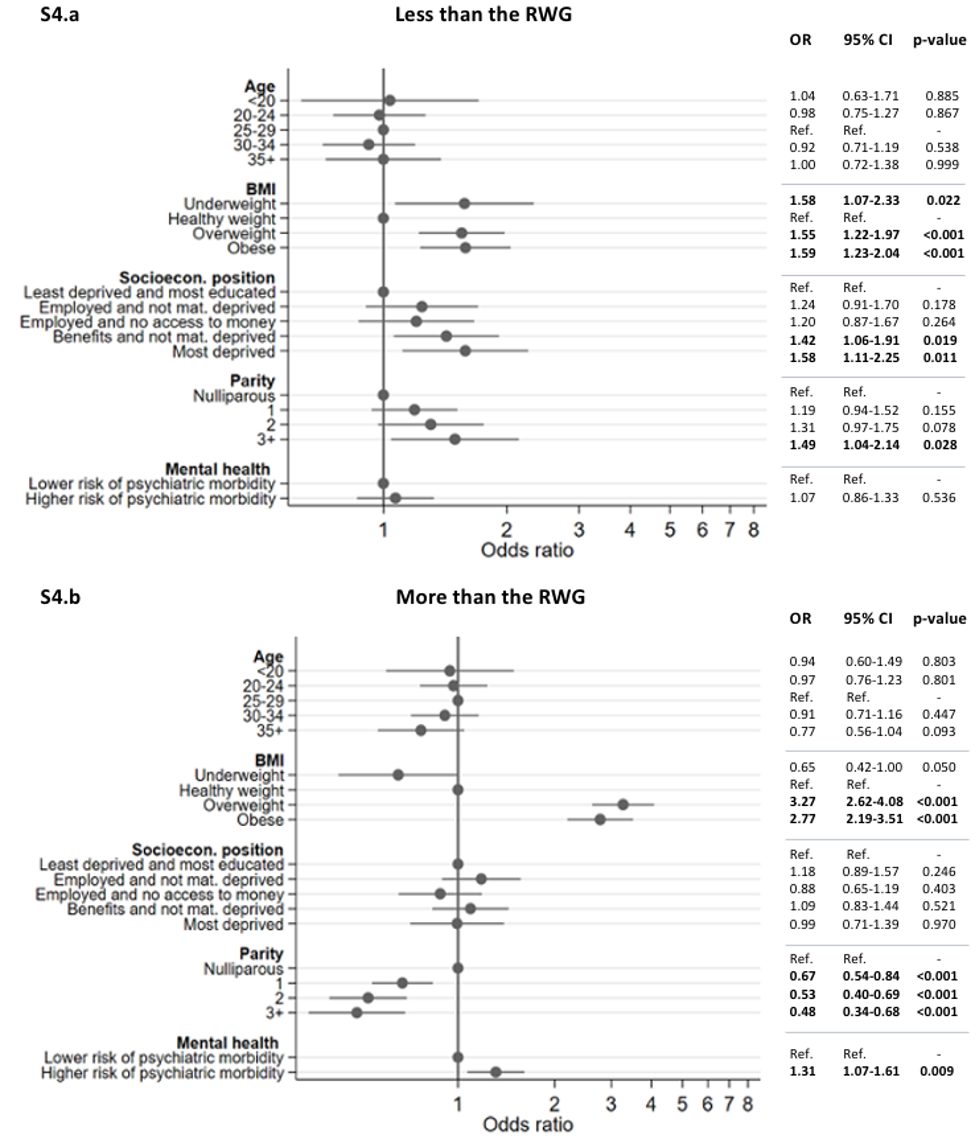

Supplement: S4 Fig — Results adjusted for ethnicity. (TIF) [file pone.0323278.s004.tif]

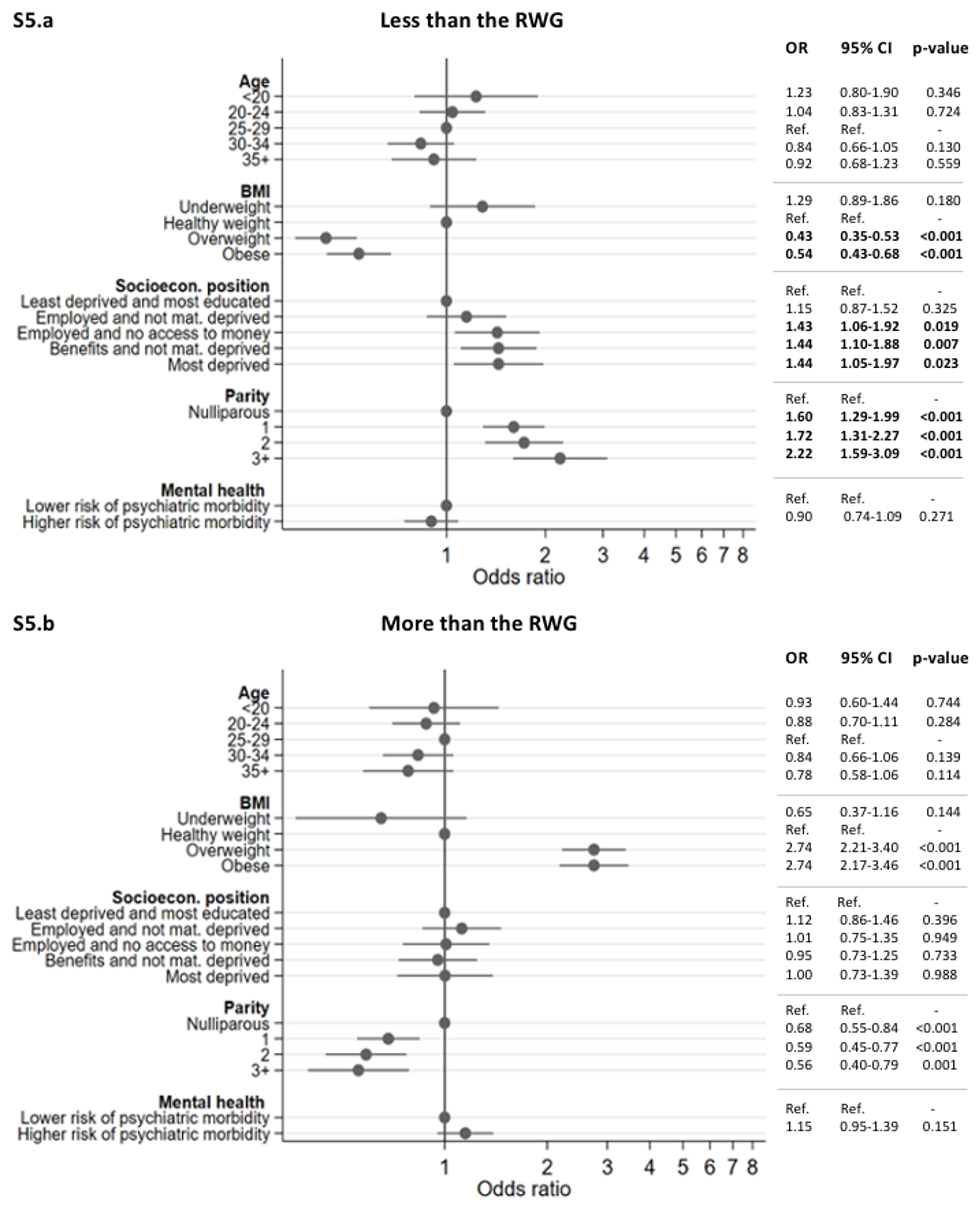

Supplement: S5 Fig — Results adjusted for ethnicity. (TIF) [file pone.0323278.s005.tif]

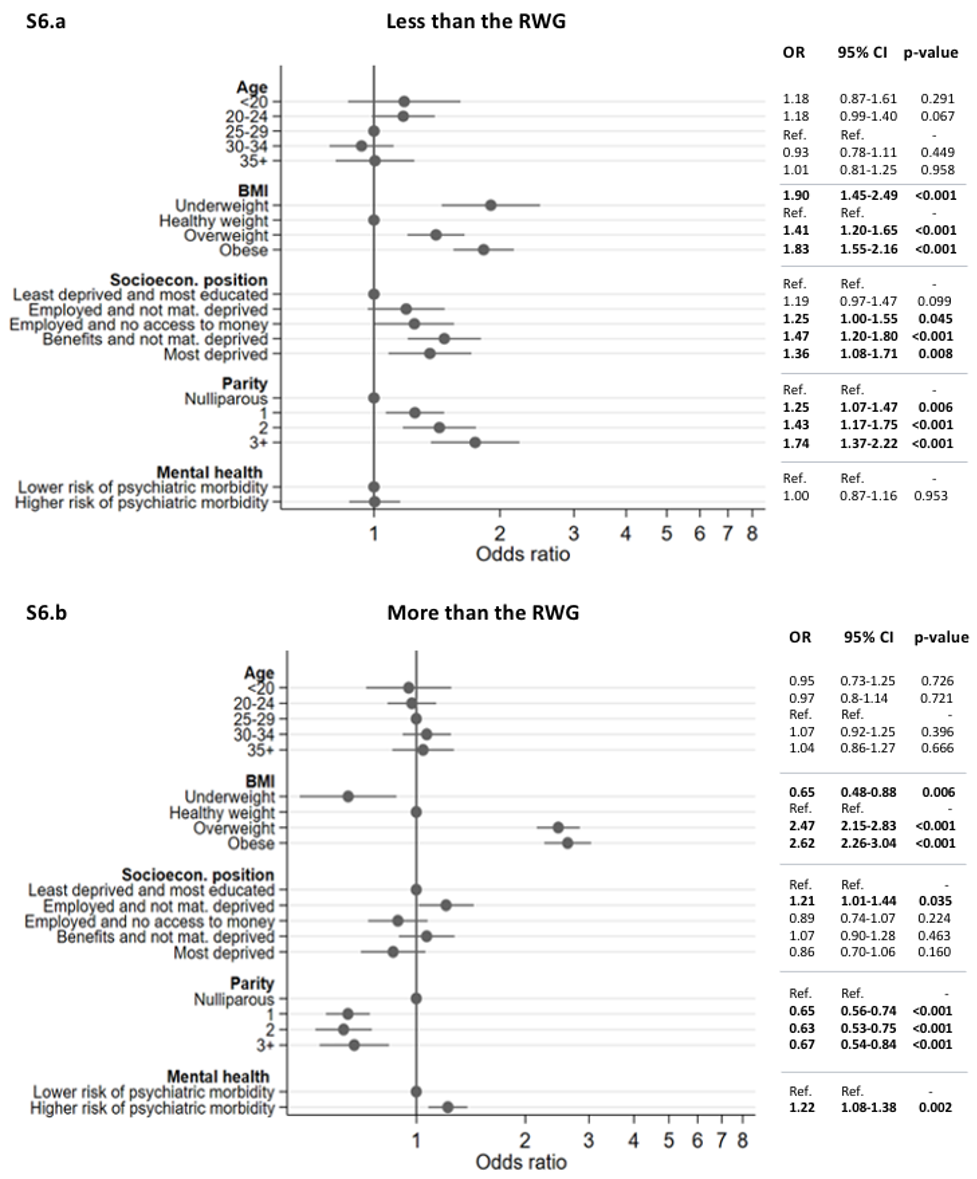

Supplement: S6 Fig — Results adjusted for ethnicity. (TIF) [file pone.0323278.s006.tif]

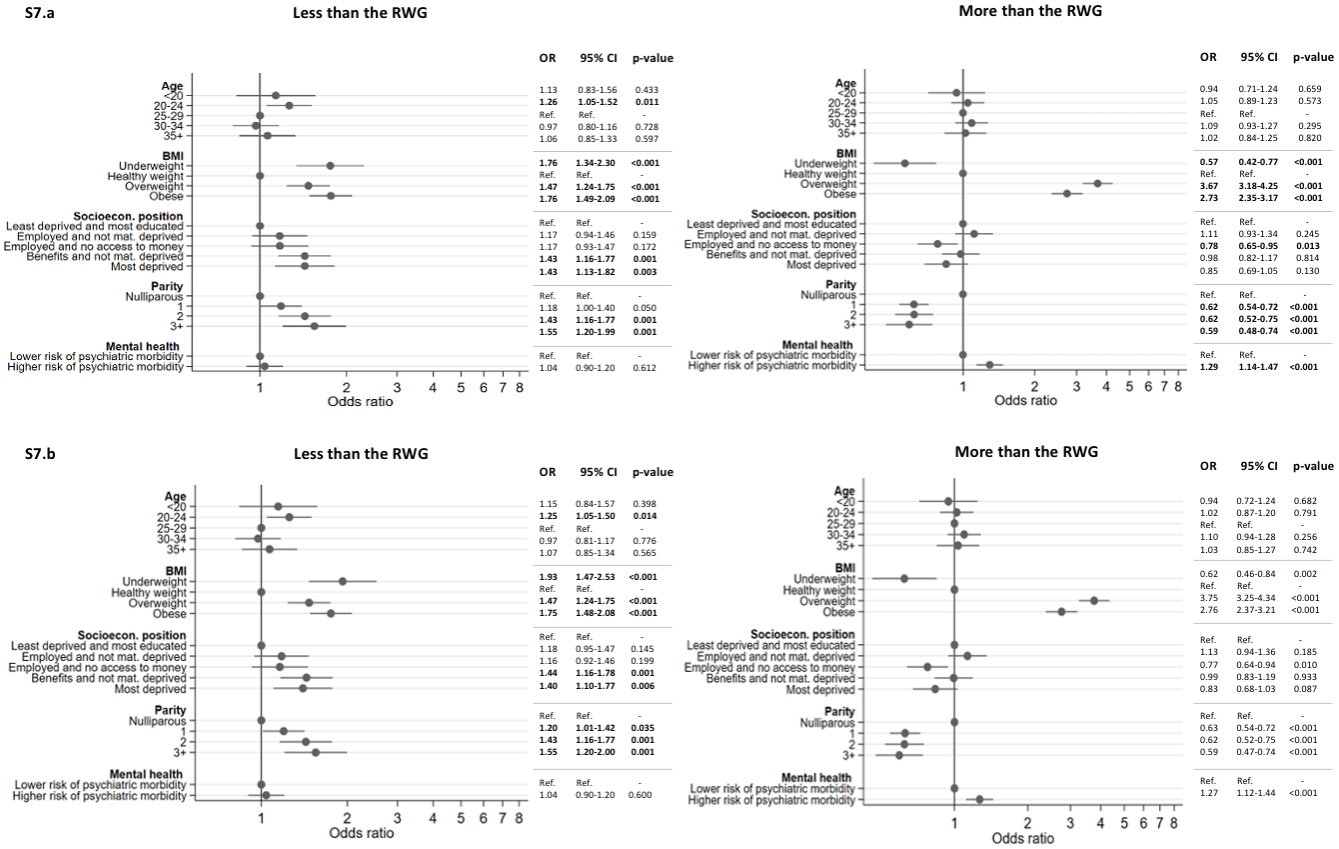

Supplement: S7 Fig — Results adjusted for ethnicity. S7.a corresponds to the addition of 0.5 kg to weight at first antenatal appointment and setting of gestational age for 14 weeks for participants with first antenatal appointment before 8 weeks. S7.b corresponds to the addition of 2 kg to weight at first antenatal appointment and setting of gestational age for 14 weeks for participants with first antenatal appointment before 8 weeks. (TIF) [file pone.0323278.s007.tif]

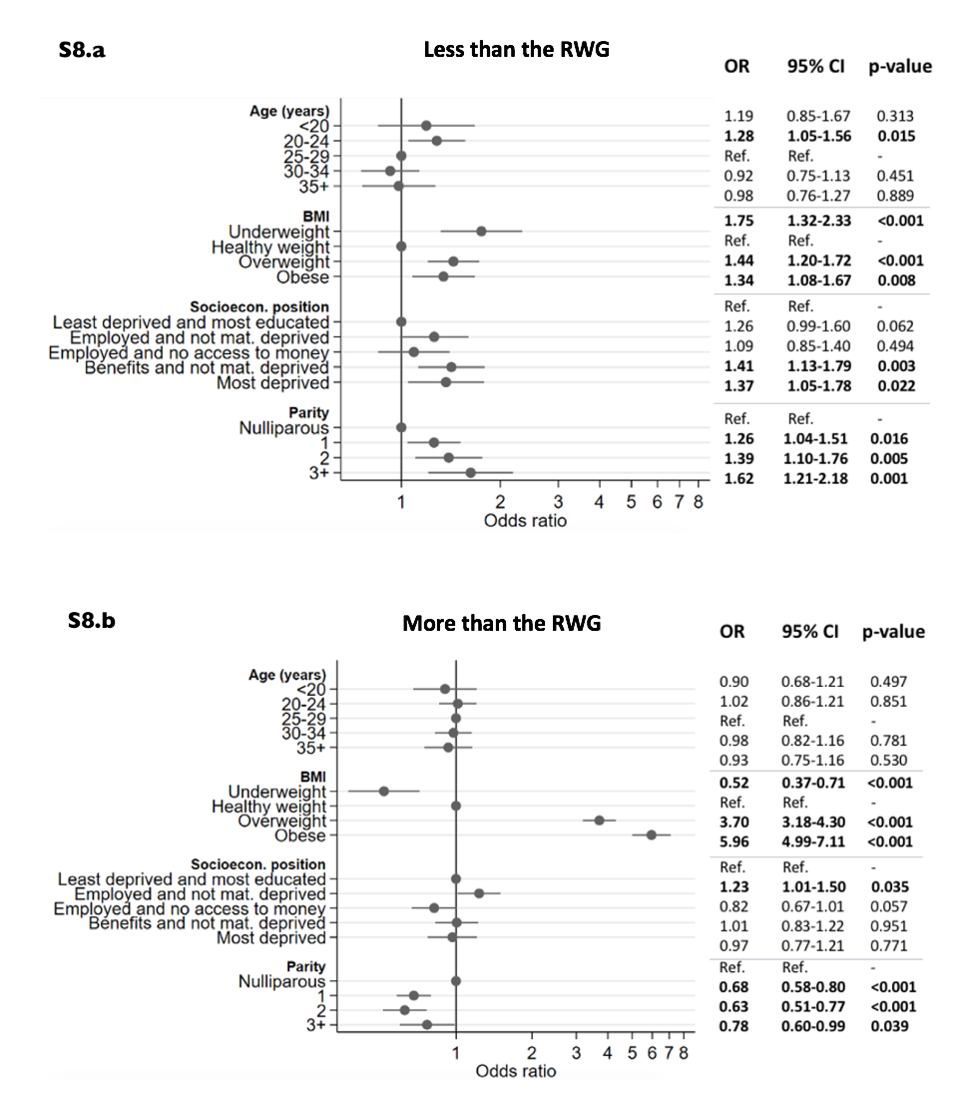

Supplement: S8 Fig — Results adjusted for ethnicity. BMI (body mass index) categories’ thresholds vary by ethnicity (S2 Table). GHQ-28 was not included in the complete case analysis due to more than 20% of missing data. (TIF) [file pone.0323278.s008.tif]
