# Supplementary material for: Determinants of gestational weight gain during pregnancy in a multiethnic UK-based population: Findings from the Born in Bradford cohort study
Source: PLoS One. 2025 May 23;20(5):e0323278. doi: 10.1371/journal.pone.0323278 (PMC12101682; doi:10.1371/journal.pone.0323278)
Supplement: S1 Table — (DOCX) [file pone.0323278.s009.docx]

**Table S1.** Institute of Medicine 2009 criteria for recommended gestational weight gain

| **Weekly rate of gestational weight gain (kg/week)** | | | |
| --- | --- | --- | --- |
|  | **Less than recommended** | **Recommended** | **More than recommended** |
| **Underweight** | <0.44 kg/wk | **0.44 to 0.58 kg/wk** | > 0.58 kg/wk |
| **Normal weight** | <0.35 kg/wk | **0.35 to 0.50 kg/wk** | > 0.50 kg/wk |
| **Overweight** | <0.23 kg/wk | **0.23 to 0.33 kg/wk** | > 0.33 kg/wk |
| **Obese** | <0.17 kg/wk | **0.17 to 0.27 kg/wk** | > 0.27 kg/wk |
| **Absolute (total) weight gain** | | | |
|  | **Less than Recommended More than**  **recommended recommended** | | |
| **Underweight** | < 12.5 kg **12.5-18 kg** > 18 kg | | |
| **Normal weight** | < 11.5 kg **11.5-16 kg** > 16 kg | | |
| **Overweight** | < 7 kg **7-11.5 kg** > 11.5 kg | | |
| **Obese** | < 5 kg **5-9 kg** > 9 kg | | |

*(Rates correspond to second and third trimester average weekly weight gain; considering a total gain of 0.5-2kg in the first trimester).*

*Adapted from IOM 2009 guideline.*
