# Supplementary material for: Determinants of gestational weight gain during pregnancy in a multiethnic UK-based population: Findings from the Born in Bradford cohort study
Source: PLoS One. 2025 May 23;20(5):e0323278. doi: 10.1371/journal.pone.0323278 (PMC12101682; doi:10.1371/journal.pone.0323278)
Supplement: S2 Table — (DOCX) [file pone.0323278.s010.docx]

**Table S2.** BMI cut-offs used for classifying pre-pregnancy BMI according to ethnic background

|  | **‘Standard’ BMI categories** | **‘Asian’ BMI categories** |
| --- | --- | --- |
| **Underweight** | < 18.5 kg/m^2^ | < 18.5 kg/m^2^ |
| **Normal weight** | 18.5–24.9 kg/m^2^ | 18.5–22.9 kg/m^2^ |
| **Overweight** | 25.0–29.9 kg/m^2^ | 23.0–24.9 kg/m^2^ |
| **Obese** | ≥ 30.0 kg/m^2^ | ≥ 25.0 kg/m^2^ |
