# Supplementary material for: Determinants of gestational weight gain during pregnancy in a multiethnic UK-based population: Findings from the Born in Bradford cohort study
Source: PLoS One. 2025 May 23;20(5):e0323278. doi: 10.1371/journal.pone.0323278 (PMC12101682; doi:10.1371/journal.pone.0323278)
Supplement: S3 Table — (DOCX) [file pone.0323278.s011.docx]

**Table S3.** Characteristics of participants included vs excluded from the complete case analysis

| **Variable** | **Total** | **Included in**  **the CCA** | **Excluded**  **from CCA** | **p-value** |
| --- | --- | --- | --- | --- |
|  | **N=7,769** | **N=7,056** | **N=713** |  |
| **Maternal age (years)** | 27.0 (24.0-31.0) | 27.0 (23.0-31.0) | 28.0 (24.0-32.0) | 0.036 |
| **Maternal age (years, categorical)** |  |  |  | 0.430 |
| <20 | 430 (5.5%) | 399 (5.7%) | 31 (4.3%) |  |
| 20-24 | 1,981 (25.5%) | 1,806 (25.6%) | 175 (24.5%) |  |
| 25-29 | 2,562 (33.0%) | 2,330 (33.0%) | 232 (32.5%) |  |
| 30-34 | 1,821 (23.4%) | 1,644 (23.3%) | 177 (24.8%) |  |
| 35+ | 975 (12.5%) | 877 (12.4%) | 98 (13.7%) |  |
| **Ethnicity** |  |  |  | <0.001 |
| White British | 3,180 (41.0%) | 2,938 (41.6%) | 242 (34.3%) |  |
| Pakistani | 3,464 (44.6%) | 3,124 (44.3%) | 340 (48.2%) |  |
| Other | 1,117 (14.4%) | 994 (14.1%) | 123 (17.4%) |  |
| **GHQ score (Mental Health)** |  |  |  |  |
| >75th centile | 1,397 (22.6%) | 1,302 (22.6%) | 95 (22.1%) | 0.800 |
| **Education** |  |  |  | 0.180 |
| <5 GCSE equivalent | 1,574 (20.6%) | 1,440 (20.4%) | 134 (23.6%) |  |
| 5 GCSE equivalent | 2,420 (31.7%) | 2,261 (32.0%) | 159 (28.0%) |  |
| A-level equivalent | 1,136 (14.9%) | 1,057 (15.0%) | 79 (13.9%) |  |
| Higher than A-level | 2,003 (26.3%) | 1,845 (26.1%) | 158 (27.8%) |  |
| Other/Foreign Unknown | 491 (6.4%) | 453 (6.4%) | 38 (6.7%) |  |
| **Currently employed** | 3,569 (46.2%) | 3,284 (46.5%) | 285 (43.1%) | 0.085 |
| **Socio-economic position** |  |  |  | 0.001 |
| Least dep and most educated | 1,542 (20.1%) | 1,435 (20.3%) | 107 (17.1%) |  |
| Employed not mat dep | 1,616 (21.0%) | 1,496 (21.2%) | 120 (19.2%) |  |
| Employed no access to money | 1,176 (15.3%) | 1,090 (15.4%) | 86 (13.7%) |  |
| Benefits but coping | 2,187 (28.5%) | 2,001 (28.4%) | 186 (29.7%) |  |
| Most deprived | 1,161 (15.1%) | 1,034 (14.7%) | 127 (20.3%) |  |
| **Marital status** |  |  |  | 0.310 |
| Married and living with partner | 5,162 (67.0%) | 4,710 (66.8%) | 452 (69.3%) |  |
| Not married and living with partner | 1,392 (18.1%) | 1,288 (18.3%) | 104 (16.0%) |  |
| Not living with partner | 1,154 (15.0%) | 1,058 (15.0%) | 96 (14.7%) |  |
| **IMD Quintiles** |  |  |  | 0.120 |
| 1 | 2,802 (36.3%) | 2,537 (36.0%) | 265 (39.9%) |  |
| 2 | 2,081 (27.0%) | 1,908 (27.0%) | 173 (26.1%) |  |
| 3 | 1,497 (19.4%) | 1,365 (19.3%) | 132 (19.9%) |  |
| 4 | 1,065 (13.8%) | 991 (14.0%) | 74 (11.1%) |  |
| 5 | 275 (3.6%) | 255 (3.6%) | 20 (3.0%) |  |
| **Parity** |  |  |  | 0.021 |
| Nulliparous | 3,071 (41.2%) | 2,921 (41.4%) | 150 (37.3%) |  |
| 1 | 2,176 (29.2%) | 2,064 (29.3%) | 112 (27.9%) |  |
| 2 | 1,258 (16.9%) | 1,189 (16.9%) | 69 (17.2%) |  |
| 3+ | 953 (12.8%) | 882 (12.5%) | 71 (17.7%) |  |
| **Smoked during pregnancy** | 1,214 (15.7%) | 1,121 (15.9%) | 93 (14.1%) | 0.230 |
| **Previous hypertension** | 62 (0.8%) | 58 (0.8%) | 4 (0.9%) | 0.930 |
| **Baseline BMI (categorical)** |  |  |  | 0.230 |
| Underweight | 337 (4.3%) | 301 (4.3%) | 36 (5.0%) |  |
| Healthy weight | 2,957 (38.1%) | 2,710 (38.4%) | 247 (34.6%) |  |
| Overweight | 2,413 (31.1%) | 2,180 (30.9%) | 233 (32.7%) |  |
| Obese | 2,062 (26.5%) | 1,865 (26.4%) | 197 (27.6%) |  |
| **Baseline BMI (kg/m^2^)** | 24.9 (21.9-29.0) | 24.9 (21.9-28.9) | 25.4 (22.1-29.3) | 0.180 |
| **Gestational weight gain (categories)** |  |  |  | 0.051 |
| Less than the RWG | 1,577 (20.3%) | 1,445 (20.5%) | 132 (18.5%) |  |
| Within the RWG | 1,743 (22.4%) | 1,558 (22.1%) | 185 (25.9%) |  |
| More than the RWG | 4,449 (57.3%) | 4,053 (57.4%) | 396 (55.5%) |  |
