# Supplementary material for: Determinants of gestational weight gain during pregnancy in a multiethnic UK-based population: Findings from the Born in Bradford cohort study
Source: PLoS One. 2025 May 23;20(5):e0323278. doi: 10.1371/journal.pone.0323278 (PMC12101682; doi:10.1371/journal.pone.0323278)
Supplement: S1 File — (DOCX) [file pone.0323278.s012.docx]

**Section S1. Method for deriving a GHQ-28 binary variable**

The general health questionnaire-28 (GHQ-28) is a tool that aims to screen and identify individuals who are “likely to have or to be at risk of developing psychiatric disorders” [44]. It is comprised of 28 items, each with four possible answers, and assesses information on somatic symptoms, anxiety/insomnia, social dysfunction and severe depression [44]. There are different methods for calculating a final score. In the present study, responses were graded according to the ‘0-0-1-1’ approach (also referred to as the binary method), which yields scores from zero to 54. Higher scores reflect impaired mental health, and different cut-offs have been used in the literature as a threshold for psychiatric morbidity. In the present study, we used cut-offs previously defined by Prady et al [24], who previously studied the application of GHQ-28 among pregnant women in the Born in Bradford cohort. They calculated the 75^th^ centile of the final score within eight ethno-language groups and selected this as the threshold for psychological distress for each group. For example, for White British women, a score of 8 was considered as the cut-off. Meanwhile, this was set as 10, 7 or 8 for Pakistani women who completed the questionnaire in English, Mirpuri or Urdu, respectively. Cut-offs for other groups can be found in the original publication [24].

Following their approach, in the present study participants with missing data for more than 4 items had their final score set to missing, due to considerations that this might represent an incomplete understanding of the questionnaire *per se*. It is important to note that since there were a few participants belonging to ethnicities with very few participants, Pardy’s study did not specify cut-offs for these groups due to insufficient data to calculate the within-group 75^th^ centile. Therefore, such participants also had their final score set to missing. We did not assess the subscales of the questionnaire because another study performed on the BiB cohort concluded that there was insufficient evidence of the appropriateness of using these psychometric subscales among pregnant women in this population [45].

**Section S2. Sensitivity Analyses**

*Sensitivity analysis 1: excluding participants with gestational diabetes mellitus*

The relationship between gestational diabetes mellitus (GDM) and gestational weight gain might be bidirectional. Studies have shown that GWG above recommendations might be a risk factor for GDM [2]. On the other hand, GDM might lead to macrosomia and polyhydramnios (increased amniotic fluid), which could potentially impact maternal weight. Additionally, women diagnosed with GDM are provided specific counseling on diet and exercise, and a proportion of them are given medications that affect weight gain (such as insulin and metformin). We deemed it appropriate to conduct a sensitivity analysis excluding patients with GDM due to the potential impact of the aforementioned particularities of this subset of participants. Out of the 7,056 women included in the final model, there were 664 (9.4%) participants with gestational diabetes mellitus. Results shown in Fig S3.

*Sensitivity analysis 2: Restriction of analysis to post-recruitment weight gain*

At the time of recruitment (˜26-28 weeks), participants responded to a questionnaire that included information on demographics, socioeconomic factors, lifestyle, and physical and mental health. Since the average weekly rate of weight gain was calculated from the first antenatal visit (˜11 weeks) onwards, at the time of questionnaire administration a relevant fraction of weight gain would have been attained (accounting for weight gained from the first antenatal visit until recruitment). Considering that the relationship between some of the information assessed through the questionnaire and GWG could be susceptible to reverse causation, we performed a sensitivity analysis restricting the investigation to weight attained after the questionnaire was administered. Post-recruitment weekly rate of GWG was calculated as the difference between the third trimester weight measurement and recruitment weight, divided by the number of weeks between those two time points. Since weight at recruitment corresponded to the last recorded weight for a subset of the participants, this analysis included only 3,525 women. Results shown in Figure S4.

*Sensitivity analysis 3: using categories of absolute (total) weight gain over pregnancy as the outcome*

The IOM set guidelines for both weekly rate of weight gain and for overall absolute (total) weight gain throughout pregnancy. The assessment of absolute (total) GWG is most commonly used in the literature. It refers to the absolute difference between the last weight measurement taken during pregnancy (close to birth) and pre-pregnancy weight [19]. In order to make findings more comparable to other studies and to overcome some of the limitations of using the average weekly rate, we conducted a sensitivity analysis using categories of total weight gain as the outcome. Unfortunately, it was not possible to obtain total weight gain for the whole sample due to an important limitation in the number of participants with late third trimester weight measurement. Therefore, the analysis was restricted to the 3,271 participants with a late third trimester measurement (≥ 36 weeks of gestational age). The proportions of participants with absolute (total) GWG below, within and above recommendations were 32.0%, 37.8% and 30.3%, respectively. Being overweight or obese at the initiation of pregnancy was associated with decreased odds of experiencing ‘less than the RWG’ in this sensitivity analysis (Fig. S5). To assess the impact of selection bias from excluding those without late third trimester weight, we repeated the main analysis with average weekly weight gain restricted to the same subgroup and the results did not differ to those in the main analysis (results not shown).

*Sensitivity analysis 4: using standard (‘Western’) BMI cut-offs for all participants*

The IOM 2009 guidelines categorize BMI according to WHO standard criteria for Western populations (S2 Table). However, the World Health Organization recognizes that the classical cut-offs used to define overweight and obesity “might not be appropriate for some populations in the Western Pacific Region” [20], because morbidity attributable to obesity occurs at lower BMI levels in these groups. Thus, the concept of Asian-Pacific BMI thresholds (S2 Table) was introduced.

Considering that more than 40% of the participants in the present study were of South-Asian origin, a misplaced categorization of their BMI could potentially lead to relevant levels of ascertainment bias in the definition of one of the main determinants under investigation (BMI) and the outcome (since categories of weight gain appropriateness are based on baseline BMI). In the main analysis of the present study, Asian-Pacific cut-offs were used to define the BMI of women from South-Asian backgrounds. In order to explore the potential impact of this approach, a sensitivity analysis using standard BMI irrespective of the ethnical background was conducted. In this context, the proportion of women in the study with underweight, healthy, overweight and obese BMI were 4.3%, 46.4%, 28.9% and 20.5%, respectively. Weight gain was deemed ‘less than the RWG’ for 26.3%, ‘within the RWG’ for 25.5% and ‘more than the RWG’ for 48.3% of women in the sensitivity analysis. In spite of these differences, there were no changes in the direction of the relationship between potential risk factors and GWG, although there were some changes in the magnitude of the effects (Fig S6).

*Sensitivity analysis 5: Correcting for presumed first trimester weight gain*

As previously mentioned, the recommended weekly weight gain proposed by IOM 2009 guideline refers to the second and third trimester [19]. Total weight gain during the first trimester is assumed to be between 0.5 and 2kg. In the present study, the median week at first antenatal appointment was 11 weeks, which is close to the end of the first trimester. Therefore, calculating the average weekly GWG from the first antenatal appointment was considered reasonable, and this approach has been previously used in other studies [16]. Nevertheless, since a small fraction of participants had an early first antenatal appointment (before 8 weeks), a sensitivity analysis with a correction for presumed first trimester weight gain was performed. We assessed the effect of adding 0.5kg and then 2kg to the weight recorded at the first antenatal appointment for this subset of participants, while changing the gestational age at measurement to 14 weeks. There was some attenuation of the effect of BMI on excessive weight gain, but the direction and significance of the relationship between determinants and GWG was not affected by these corrections (Fig S7).

*Sensitivity analysis 6: Complete case analysis*

A complete case analysis was performed as a sensitivity analysis (Fig S8) to compare results with the imputed model. There were 713 participants excluded from the analysis due to missing data items. The characteristics between the included and excluded can be seen in Table S3.

**Supporting References**

44. Sterling M. General Health Questionnaire – 28 (GHQ-28). Journal of Physiotherapy. 2011;57(4):259.

45. Prady SL, Miles JN, Pickett KE, Fairley L, Bloor K, Gilbody S, et al. The psychometric properties of the subscales of the GHQ-28 in a multi-ethnic maternal sample: results from the Born in Bradford cohort. BMC Psychiatry. 2013;13(1):55.
